# Successful Video-Assisted Thoracoscopic Removal of a Chest Tube Protruding Deep Into the Pulmonary Hilum

**DOI:** 10.7759/cureus.24406

**Published:** 2022-04-23

**Authors:** Mikito Suzuki, Hirotoshi Horio, Reiko Shimizu, Toshiyuki Shima, Masahiko Harada

**Affiliations:** 1 Department of Thoracic Surgery, Tokyo Metropolitan Cancer and Infectious Diseases Center Komagome Hospital, Tokyo, JPN

**Keywords:** lung resection, video-assisted thoracoscopic surgery, tube thoracostomy, pulmonary hilum, empyema, chest tube

## Abstract

Tube thoracostomy is an invasive procedure frequently used to drain pleural fluid collections or to manage pneumothorax, wherein the lungs commonly sustain trauma. In some cases, deep pulmonary hilar injuries are managed by anatomical lung resections. A deep hilar injury with the chest tube protruding into the lung parenchyma is a rare complication of tube thoracostomy. We report the case of a patient with tube thoracostomy-related deep pulmonary laceration treated using video-assisted thoracic surgery (VATS).

A 74-year-old man with a left-sided pneumonia-associated empyema underwent tube thoracostomy for drainage of intrathoracic purulent collection at another hospital; however, chest radiography and computed tomography (CT) revealed intrusion of the 22 Fr chest tube into the left lower lobe parenchyma for approximately 10 cm toward the pulmonary hilum, with the chest tube tip located near the left main bronchus and pulmonary artery. Although no massive intrapulmonary hemorrhage, pneumothorax, or pneumomediastinum was observed, multiple pyothoracic cavities were present. He was transferred to our hospital the following day in a hemodynamically stable condition. The next day, he underwent both surgical chest tube removal and decortication for empyema. Owing to the worsening of his physical condition and due to prolonged severe inflammation and lack of appetite, without any sign of bleeding or chest tube air leak, a two-port VATS with sparing of the lung parenchyma was attempted. After decortication, the penetrating chest tube was slowly removed. No hemorrhage or air leaks were observed at the site of penetration, requiring no sutures or dressing. Following his uneventful postoperative course, he was discharged on day 9.

In selected cases, anatomic lung resection can be avoided, even for deep hilar injuries, depending upon the degree of intrapulmonary hemorrhage and the presence of air leak from the chest tube, and the CT scan findings.

## Introduction

Tube thoracostomy is an invasive procedure, and the most common complication of this procedure is lung injury [1,2]. The treatment of tube thoracostomy-induced lung injuries ranges from conservative treatment to lung resections depending on the extent of the injury and the patient’s condition [3,4]. Herein, we report the successful removal of a chest tube protruding deep into the pulmonary hilum without resorting to lung resection using a video-assisted thoracic surgery (VATS) approach.

## Case presentation

**Figure 1:**
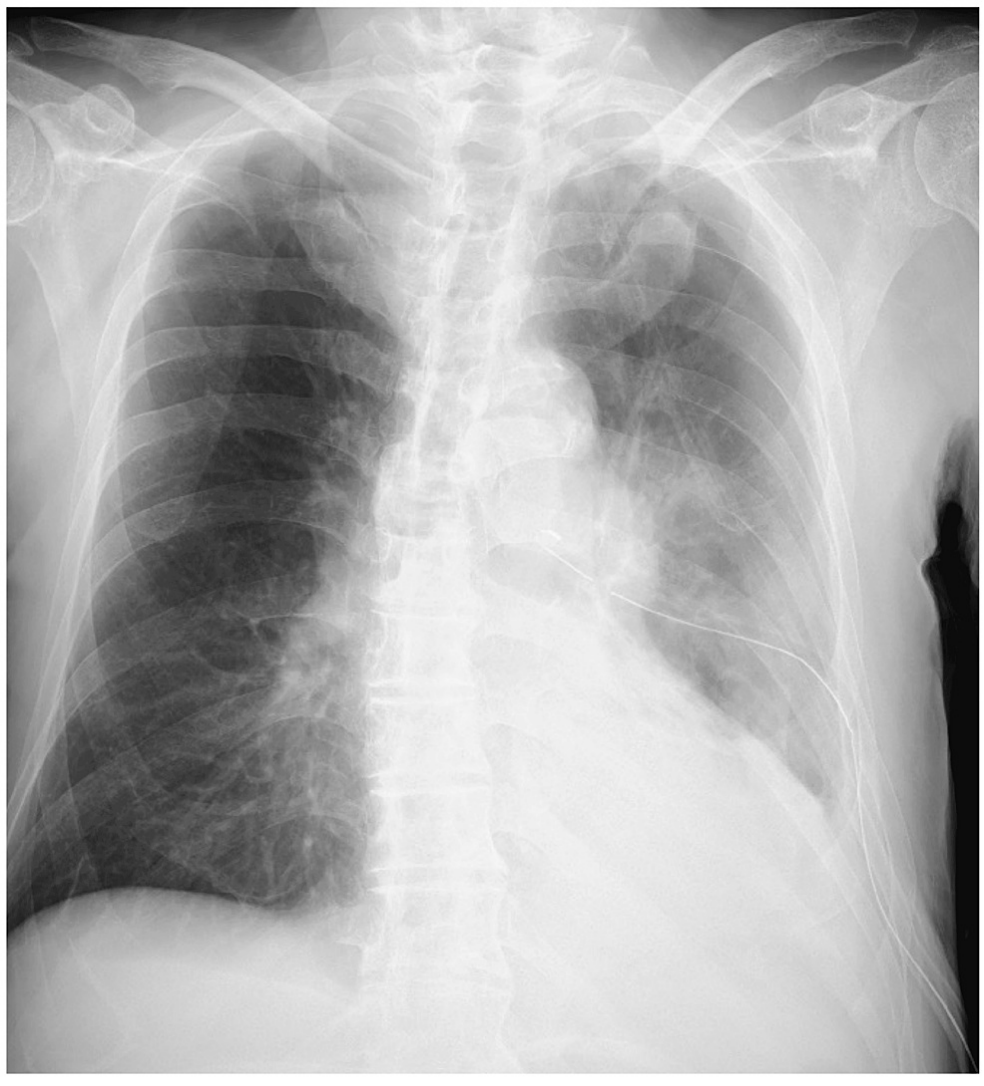
Preoperative chest radiography finding The chest tube was inserted through the left seventh intercostal space. Hazy opacities and pleural effusion were observed in the left middle and lower lung zones.

A 74-year-old man with a three-week history of malaise, anorexia, and fever presented with left-sided pneumonia-associated empyema diagnosed based on thoracentesis, laboratory tests, and radiological findings at another hospital. A 22-Fr chest tube was inserted using the trocar technique through the left seventh intercostal space by pulmonologists at the other hospital, followed by drainage of a small amount of pleural fluid. Thereafter, no fluctuation was noted in the level of the water-sealed chamber. From a chest radiography finding, suspecting chest tube malposition, and computed tomography (CT) were performed (Figure 1).

**Figure 2:**
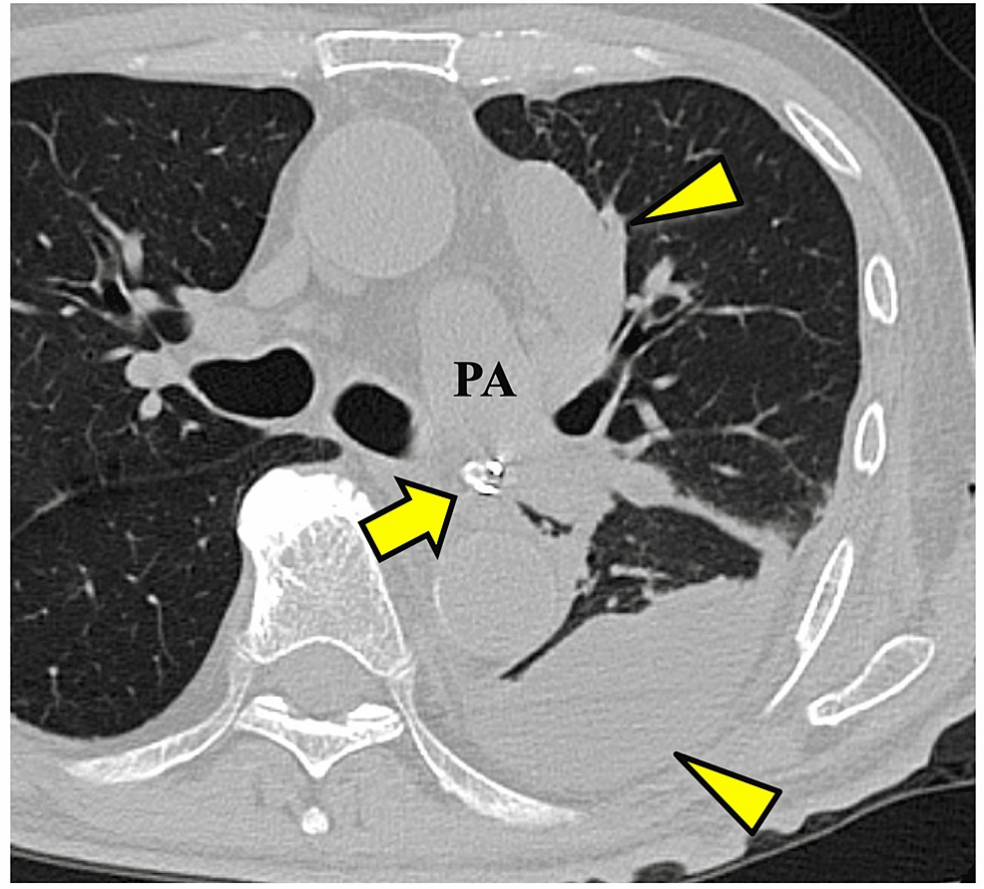
Preoperative chest computed tomography finding The chest tube protruding into the left lower lobe. The tip of the chest tube is placed near the left main bronchus and pulmonary artery (arrow). Multiple pyothoracic cavities are present (arrowhead). PA: pulmonary artery

**Figure 3:**
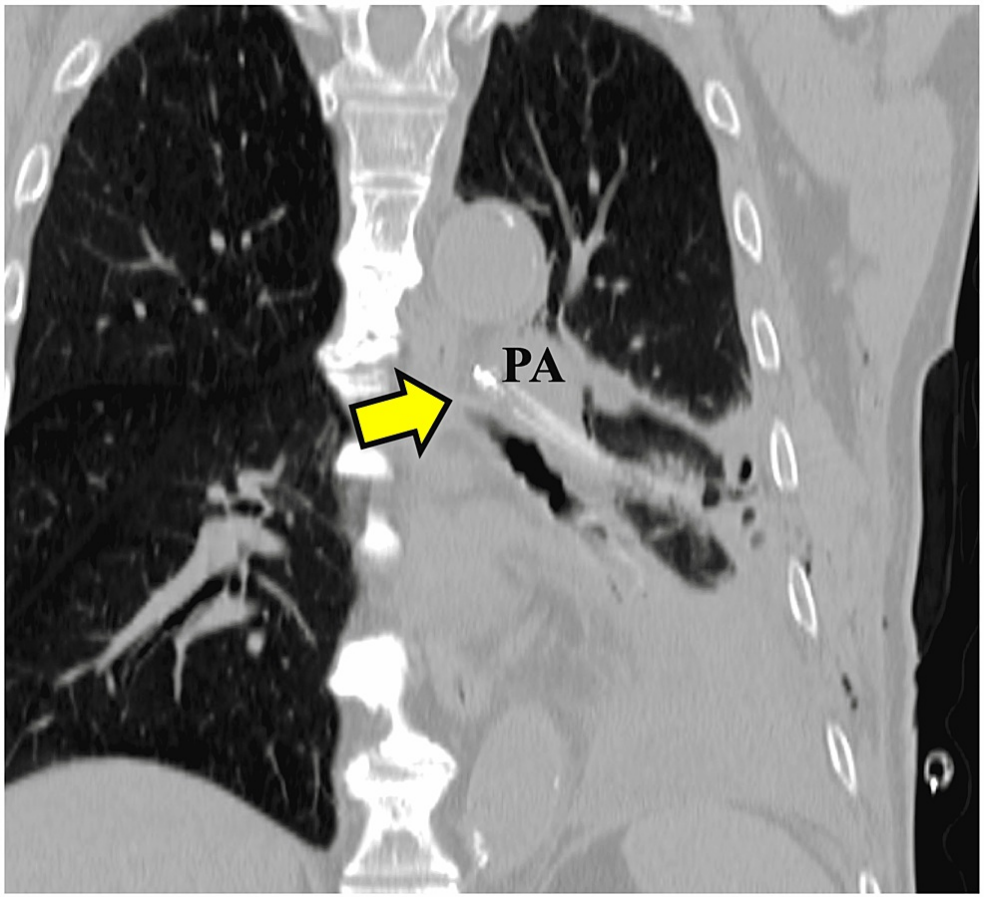
Preoperative coronal computed tomography The chest tube has migrated approximately 10 cm into the left lower lobe (arrow). No massive intrapulmonary hemorrhage, pneumothorax, or pneumomediastinum is observed. PA: pulmonary artery

CT revealed approximately 10-cm intrusion of the chest tube into the left lower lobe parenchyma, with its tip located near the left main bronchus and main pulmonary artery (Figures 2, 3).

 

Minor pulmonary contusion was observed around the chest tube, with no evident pneumothorax or pneumomediastinum. The patient was asymptomatic and hemodynamically stable; however, his performance status worsened from 0 to 2 due to a severe inflammatory response to the intrapulmonary chest tube and lack of appetite. He was transferred to our hospital the following day for management of empyema and iatrogenic lung laceration. His past medical history included hypertension. At presentation, his body temperature was 39.7°C and pulse rate was 87 beats/min. His blood pressure and oxygen saturation on room air were normal. Laboratory test results showed increased white blood cell count (21,500 cells/μL) and C-reactive protein levels (35.5 mg/dL) and decreased albumin (1.9 g/dL) and hemoglobin (10.2 g/dL) levels due to inflammation and malnutrition. No bleeding or air leak was observed from the chest tube.

With the laboratory parameters showing ongoing infection and chest CT showing multiple pyothoracic cavities, continuing conservative management would have been detrimental to the patient’s condition. Therefore, surgery was planned on the following day for decortication of empyema and subsequent chest tube removal. Based on his poor performance status and laboratory evidence of ongoing infection and pyothorax and with no sign of air leak or hemorrhage, a two-port VATS approach was employed in an attempt to avoid anatomical lung resection.

**Figure 4:**
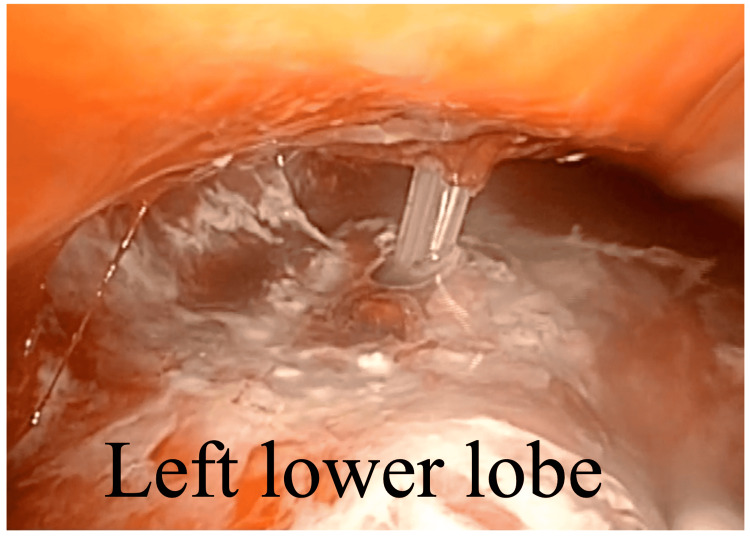
Intraoperative findings of a chest tube protruding into the left lower lobe observed after decortication

A 2-cm skin incision was made in the eighth intercostal space on the midaxillary line and in the ninth intercostal space on the posterior axillary line. The pleural cavity was completely adhered to owing to the empyema. We decorticated the pyothoracic cavity extending from the diaphragm toward the apical portion and found that the chest tube had punctured the superior segment of the left lower lobe (Figure 4).

**Figure 5:**
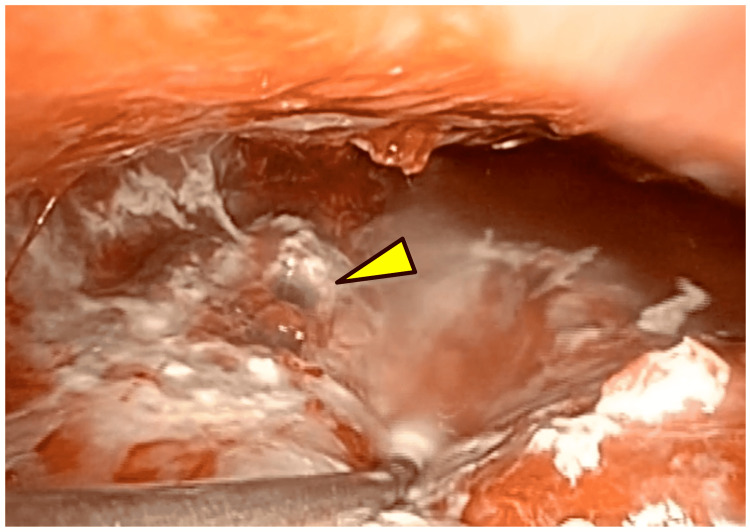
After removal of the chest tube, no hemorrhage or air leak was observed from the punctured site (arrowhead).

After removing the surrounding adhesions, we slowly withdrew the chest tube. No hemorrhage or air leak was observed; therefore, no sutures or dressing were required (Figure 5).

**Figure 6:**
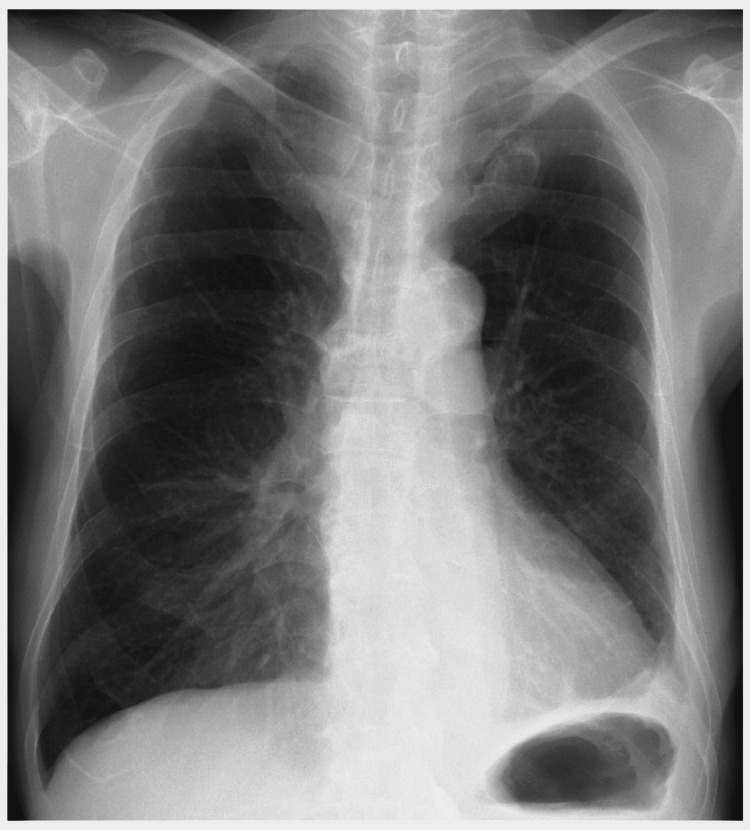
Chest radiograph finding after surgery The chest tube intruding into the left lung parenchyma was successfully removed, and pyothoracic cavities were decorticated.

The operative time was 64 min, and blood loss was minimal. His postoperative course was uneventful. The newly inserted chest tube was removed on postoperative day 6, and he was discharged on postoperative day 9. Six months after the surgery, the patient had progressed without complications (Figure 6).

## Discussion

Tube thoracostomy is frequently performed for drainage of pleural fluid collections or pneumothorax. The lung is the most commonly injured organ during tube thoracostomy, and the complication rate of intraparenchymal tube placement has been reported to be as high as 9% [1,2]. Although pulmonary hilar injuries due to tube thoracostomy without protrusion of the lung parenchyma under lung collapsed condition due to pneumothorax or pleural effusion were rarely reported [5], such injuries with protrusion into the lung parenchyma are extremely rare. Patients with decreased lung compliance, consolidation of the underlying lung parenchyma, or tight pleural adhesions are at an increased risk of lung laceration [3]. In our patient, tight pleural adhesions due to empyema were observed. The inadvertent insertion of the chest tube - at a short distance of 1 cm from the lung parenchyma - was partially responsible for the lung laceration. Using the trocar technique for tube thoracostomy placement is also a risk factor for causing penetrative lung injuries [6], consistent with the finding in our case. Furthermore, we should recognize the diagnosis of protrusion of the lung parenchyma by chest radiography was often unreliable because distinguishing intraparenchymal tube placement from pleural cavity placement was difficult in two-dimensional images, especially with preexisting pulmonary disease [4].

Tractotomy has been used for deep pulmonary penetrating injuries as a lung parenchyma-sparing technique [7,8]. In tractotomy, which has a mortality rate of 27%, the penetrated part of the lung is incised, and the bleeding point, pulmonary fistula, and margins are sutured [7]. However, tractotomy is not indicated for hilar injuries, and anatomical lung resection is performed as the last resort [7,8]. In our case, although no persistent hemorrhage or air leaks were present before surgery, re-bleeding and air leak from the deep hilum could develop due to the stimulus of chest tube removal; hence, surgical removal was indicated. As the patient’s performance status worsened due to a prolonged inflammatory state and lack of appetite, highly invasive lobectomy was avoided as it would be challenging due to empyema-related extensive adhesions. Furthermore, lower lobectomy and hypoalbuminemia have been reported as risk factors for resultant bronchopleural fistula post-lobectomy [9]. Based on the chest tube and CT findings, we attempted to avoid lobectomy. Since there were no air leaks or bleeding at the site of chest tube penetration, and good lung compliance was achieved after re-ventilation, sutures, and dressing were not required. Air leaks were not observed after chest tube removal because there was, fortunately, no central airway injury. Distal airway leakage stopped due to the pressure of the inserted chest tube, which was left in situ for >2 days.

Systematic air embolism (SAE) is a rare but lethal complication of lung injuries. The pulmonary vein and airways are simultaneously damaged, inducing air entrapment in the injured pulmonary vein [10]. The presence of air shunting, pneumothorax, lung contusions, and positive pressure ventilation can be risk factors for SAE, as there is a continuous flow of air in the vicinity of the systemic arterial circulation [11]. In our case, CT scan images post lung injury showed minor lung contusion, and no persistent intrapulmonary hemorrhage or air leak was observed for two days preoperatively. Thus, the risk of the occurrence of SAE after one-lung ventilation was deemed to be low, making the VATS approach preferable. However, late-onset SAEs have rarely been reported in penetrating chest traumas. Positive pressure ventilation could be a trigger for late-onset SAE. Therefore, one-lung ventilation during general anesthesia is recommended [10]. In cases of sudden hemodynamic instability and neurological symptoms in patients with lung injury on mechanical ventilation, SAE should be suspected.

## Conclusions

Deep hilar injuries by an intraparenchymal protrusion of the chest tube during tube thoracostomy are rare and can cause uncontrollable intrapulmonary hemorrhage and air leaks. Case reports on the management of deep hilar injuries without resorting to anatomical lung resections are limited; therefore, our study findings can serve as a reference when managing such cases in clinical settings.

We reported a case of deep lung laceration resulting from tube thoracostomy that was treated using a VATS approach without lung resection. The degree of hemorrhage, air leak from the chest tube, and CT findings could help choose an appropriate surgical approach.
